# Péritonite primitive: entité réelle mais de diagnostic difficile

**Published:** 2012-07-24

**Authors:** Rajae Yamou, Mohammed Najih, Mohamed Absi, Mohamed Ouanani, Mahjoub Echerrab, Hassan EL Alami, Mohamed Amraoui, Abdelkader Errougani, Mohamed Rachid Chkoff

**Affiliations:** 1 Service des urgences chirurgicales viscérales, Hôpital Ibn Sina, Rabat, Maroc

**Keywords:** Diagnostic, péritonite primitive, traitement, Diagnosis, primitive peritonitis, treatment

## Abstract

Les péritonites primitives sont rares chez des patients sans facteurs de risques. Elles simulent les péritonites secondaires. C'est pourquoi leur diagnostic ne peut être que peropératoire. Nous rapportons deux cas de péritonites primitives dans le but de discuter leurs aspects diagnostique et thérapeutiques.

## Introduction

La péritonite primitive (PP), encore appelée idiopathique ou spontanée, est définie comme un processus infectieux touchant la cavité péritonéale et ne provenant ni d'une perforation viscérale, ni d'un processus inflammatoire survenant dans la cavité abdominale ou à son voisinage, ni d'une plaie pénétrante [1]. En excluant de ce cadre les tuberculoses péritonéales révélées selon un mode aigu ainsi que les ascites infectées en particulier chez les cirrhotiques et les insuffisants rénales chroniques sous dialyse péritonéale, ces PP demeurent exceptionnelles. Leur intérêt particulier provient du fait que leur traitement est toujours chirurgical vue la constante incertitude du caractère primitif qui reste exceptionnel surtout en absence de contexte évocateur tel que les circonstances sus-citées. L'objectif de ce travail est de démontrer l'existence de cette entité et de rappeler les difficultés diagnostiques et la prise en charge thérapeutique des péritonites primitives.

## Patients et observations

### Observation 1

Patient de 57 ans, diabétique sous insuline depuis 3 ans consultait pour des douleurs abdominales diffuses avec des vomissements évoluant depuis 3 jours dans un contexte fébrile. L'examen clinique a trouvé un patient conscient dont l’état général était conservé, fébril à 39°;C, l'examen abdominal a trouvé une contracture abdominale généralisée. La recherche d'acétone dans les urines était négative et la glycémie capillaire à l'admission était à 1,86g/l. Les clichés d'abdomen sans préparation et de radiographie thoracique ne montraient pas de pneumopéritoine. L’échographie et la TDM abdominales trouvaient un épanchement intrapéritonéal de moyenne abondance impur, sans visualisation de l'appendice. Le bilan biologique a montré une hyperleucocytose à 14000 /mm^3^, une élévation de la CRP à 80mg/l et une fonction rénale normale. Le patient a été opéré pour le diagnostic d'une péritonite aiguë généralisée. La voie d'abord était une laparotomie médiane à cheval sur l'ombilic. A l'exploration, on a trouvé un épanchement trouble de moyenne abondance avec quelques fausses membranes. L'appendice était d'aspect normal, et il n'y avait pas de perforation d'organe creux. On a réalisé un prélèvement pour analyse bactériologique, une appendicectomie de principe, une toilette péritonéale abondante avec du sérum salé 9 1/1000 enfin un drainage large par des lames de delbet et un drain de Redon. Le patient a été mis sous antibiothérapie probabiliste à large spectre à base d'amoxicilline protégée associée à une aminoside et métronidazole en attendant le résultat bactériologique avec surveillance de ses chiffres glycémiques. Les suites opératoires étaient simples. Le germe identifié était un bacille gram négatif (BGN) *Escherchia coli* résistant à l'amoxicilline protégée mais sensible aux autres béta lactamines en particulier les céphalosporines de 3^ème^ génération. Le traitement antibiotique a été ajusté. Les moyens de drainage ont été enlevés le 5^ème^ jour postopératoire et le patient est sorti le lendemain. Il a été revu après une semaine. Le recul actuel est de 1 an sans anomalies.

### Observation 2

Patiente de 48 ans, ayant un antécédent de lymphoedème chronique du membre inférieur droit, présentait des douleurs abdominales diffuses depuis 2 jours avec une fièvre. A l'admission, la patiente était consciente, fébrile à 38,8°, pesant 130 kg pour une taille de 1,58 m soit un IMC à 52kg / m^2^, l'examen abdominal a trouvé une défense abdominale généralisée. Le cliché de radiographie thoracique ne montrait pas de pneumopéritoine. L’échographie abdominale trouvait un épanchement intrapéritonéal au niveau du cul de sac de Douglas et en interanse. Le bilan biologique a montré une hyperleucocytose à 22000 /mm^3^, une élévation de la CRP à 110 mg/l et un taux normal de lipasémie.

L'exploration par une voie d'abord médiane à cheval sur l'ombilic élargie en sous ombilicale a trouvé un épanchement d'aspect purulent avec des fausses membranes. L'appendice était normal et l'examen complet de toute la cavité intrapéritonéale ne trouvait pas de perforation d'organe creux ni de foyer abcédé responsable de cette contamination. Cependant on a trouvé un kyste de l'ovaire gauche d'environ 7cm/5 cm qu'on a réséqué. On a réalisé un prélèvement pour étude bactériologique, une appendicectomie de principe, une toilette péritonéale abondante enfin un drainage large par des lames de delbet et un drain de Redon. La patiente a été mise sous antibiothérapie à large spectre à base d'amoxicilline protégée associée à une aminoside et métronidazole. Les suites opératoires étaient marquées par une infection de la paroi. Le germe trouvé était un bacille gram négatif (BGN) qui n'a pas été identifié. La patiente est sortie le 8^ème^ jour. Le traitement antibiotique a été continué pendant 10 jours. Toutefois, l'infection de la paroi a nécessité 1 mois pour qu'elle se tarisse. Le recul actuel est de 18 mois.

## Discussion

Les péritonites primitives ou dites encore spontanées sont définies comme étant des infections aigues à point de départ péritonéal survenant en l'absence de foyer septique primaire intrapéritonéal et sans solution de continuité du tube digestif [2, 3]. Si chez des patients cirrhotiques, les PP ont une prévalence de 8 à 30% [1–4], elles sont très rares chez des patients sans terrain prédisposant représentant 1 à 3% de l'ensemble des péritonites [5, 6]. Elles sont le plus souvent liées à une inoculation bactérienne mono-microbienne par voie hématogène à partir d'un foyer infectieux pulmonaire. Plus rarement la voie lymphatique, transdiaphragmatique ou par migration transmurale depuis la lumière intestinale est incriminée. Le mécanisme reste mal élucidé. Selon Salleras [7], l'hypothèse d'une thrombogénèse au niveau des veines de drainage avec fragmentation des thrombi serait à l'origine de la formation d'un essaim d'emboles septiques qui vont constituer une vascularite suppurée au niveau des différents viscères. L'embolisation des vaisseaux de la sous-séreuse du grêle entraîne la colonisation par contiguïté du péritoine. Les lésions péritonéales sont variées: inflammation péritonéale, péritonite enkystée. Comme les péritonites secondaires, elles se présentent souvent sous le même tableau clinique avec une évolution rapide, douleur spontanée et provoquée par la palpation, plutôt diffuse, sans localisation pouvant orienter vers un organe ou un autre avec contracture justifiant une laparotomie en urgence. Parfois, elles peuvent être moins bruyantes. En principe, leur traitement peut être uniquement médical à base d'antibiotiques, cependant l'exploration chirurgicale reste obligatoire vu leur diagnostic difficile afin d’éviter le risque gravissime de méconnaitre une cause sous-jacente. Néanmoins, l'absence de foyer primaire et de cause intra-abdominale évidente est responsable de deux problèmes; d'une part et vu le caractère primitif très rare qui reste un diagnostic d’élimination, le chirurgien doit réaliser une exploration minutieuse très détaillée pour ne pas passer à côté d'une cause. Le deuxième réside dans les cas opérés sous coelioscopie pour lesquels le chirurgien convertit en une laparotomie pour mieux explorer avant d'affirmer le diagnostic de péritonite primitive. Le lavage péritonéal abondant reste efficace dans toute forme de péritonite [8, 9]. Il diminue par élution le nombre de germes initialement présents dans l'abdomen, évitant ainsi la survenue d'une péritonite postopératoire. Le pronostic est fonction du terrain et du délai de mise en route du traitement adapté. L'affection est redoutable chez le malade immunodéprimé [10].

## Conclusion

Le diagnostic de péritonite primitive est quasi-impossible en préopératoire. Ainsi, dans le cadre de l'urgence la laparotomie est inévitable. Le lavage péritonéal associé à une antibiothérapie adaptée reste le traitement de choix. La difficulté pour le chirurgien étant de considérer ce diagnostic comme possible est de ne pas convertir pour chercher une cause hypothétique non vue en laparoscopie.
